# Using the nonlinear control of anaesthesia-induced hypersensitivity of EEG at burst suppression level to test the effects of radiofrequency radiation on brain function

**DOI:** 10.1186/1753-4631-3-5

**Published:** 2009-07-18

**Authors:** Tarmo Lipping, Michael Rorarius, Ville Jäntti, Kari Annala, Ari Mennander, Rain Ferenets, Tommi Toivonen, Tim Toivo, Alpo Värri, Leena Korpinen

**Affiliations:** 1 Tampere University of Technology, Pori, Finland; 2 Tampere University Hospital, Tampere, Finland; 3 Seinäjoki Central Hospital, Seinäjoki, Finland; 4 STUK, Radiation and Nuclear Safety Authority, Helsinki, Finland; 5 Faculty of Medicine, University of Tampere, Tampere, Finland

## Abstract

**Background:**

In this study, investigating the effects of mobile phone radiation on test animals, eleven pigs were anaesthetised to the level where burst-suppression pattern appears in the electroencephalogram (EEG). At this level of anaesthesia both human subjects and animals show high sensitivity to external stimuli which produce EEG bursts during suppression. The burst-suppression phenomenon represents a nonlinear control system, where low-amplitude EEG abruptly switches to very high amplitude bursts. This switching can be triggered by very minor stimuli and the phenomenon has been described as hypersensitivity. To test if also radio frequency (RF) stimulation can trigger this nonlinear control, the animals were exposed to pulse modulated signal of a GSM mobile phone at 890 MHz. In the first phase of the experiment electromagnetic field (EMF) stimulation was randomly switched on and off and the relation between EEG bursts and EMF stimulation onsets and endpoints were studied. In the second phase a continuous RF stimulation at 31 W/kg was applied for 10 minutes. The ECG, the EEG, and the subcutaneous temperature were recorded.

**Results:**

No correlation between the exposure and the EEG burst occurrences was observed in phase I measurements. No significant changes were observed in the EEG activity of the pigs during phase II measurements although several EEG signal analysis methods were applied. The temperature measured subcutaneously from the pigs' head increased by 1.6°C and the heart rate by 14.2 bpm on the average during the 10 min exposure periods.

**Conclusion:**

The hypothesis that RF radiation would produce sensory stimulation of somatosensory, auditory or visual system or directly affect the brain so as to produce EEG bursts during suppression was not confirmed.

## Background

Indications of the RF-EMF originated changes in biological systems have been researched by exposing human volunteers, animals and cells to radio frequency (RF) fields as well as by performing epidemiological studies [1-6]. The studies on the effect of exposure to RF fields on brain functions and among others on behaviour of test animals have yielded contradictory results [7-13]. In many studies the applied RF frequencies and their modulation have not corresponded to the frequencies used by commercial mobile phones. The background radiation has complicated the evaluation of the results.

Animal studies are especially useful for experiments in which the exposure level exceeds the limits for human exposure (for exposure levels, see [14]). The use of high exposure levels is an essential element of toxicological testing (e.g., safety testing of chemicals): sufficiently high exposure levels are used to find a biological response, and lower exposures are then systematically tested to find the "safe" level (taking into account possible sensitivity differences between the test animals and humans). This kind of toxicological approach has not yet been applied for systematic testing of the effects of RF fields on brain function in animals.

Animal studies are also well suited for studying the hypothesized specific effects of amplitude-modulated RF fields on the central nervous system. If specific bioeffects of amplitude-modulated fields exist (for review, see [1]), they would be especially relevant for current digital mobile phone systems such as GSM. A few studies have reported effects of modulated RF fields on animal electroencephalogram (EEG) [15-17], but systematic research on this question is lacking, and the positive findings have not been followed up. In this study anaesthetised pigs are used as test animals. The pig, with its relatively large brain size, has proven to be one of the best options as a test animal when considering effects on the brain.

During anaesthesia the EEG shows less variability compared to the waking condition when sensory-motor activity, cognitive functions and mental activity among other factors might cause the properties of the signal to fluctuate in an unpredictable manner. Anaesthesia itself modulates the properties of the EEG, however, these changes are relatively well known. We have previously shown that during burst suppression, i.e., the phase of deep anaesthesia when periods of relatively flat EEG alternate with high amplitude mixed frequency bursts, the bursts can be provoked by somatosensory, photic and auditory stimuli [18,19]. In fact, minor stimuli such as vibration [20] or light touch [21-23] readily produce bursts. Bursts can be produced by both the onset and end of a train of stimuli with the latency remaining fairly constant. Burst suppression is closely related to slow wave sleep oscillations, which develop a nonlinear control system for intermittent suppressions. Suppression segments are essentially prolonged up-states of the cortical sleep oscillation [23]. In anaesthesia with volatile agents as well as ischemic brain damage this nonlinear, on-off, control system sometimes becomes reactive to minimal perturbations. This phenomenon has been called hypersensitivity of the anaesthesia-induced comatose brain [24,25]. We therefore hypothesized that this extremely sensitive phenomenon, representing a very sensitive nonlinear control system, might be an indicator of possible RF effects.

The objective of this study was to determine the effects of radiofrequency radiation from the GSM mobile phone system on the electrical activity of a pig brain at radiation levels above those accepted for human testing. The aims were to find the threshold for observable changes in the brain's electrical activity, to document the consequences of exposure above the threshold, and to study whether there is any difference between the effects of short exposure pulses and continuous exposure. The experiments were designed with two objectives in mind: 1) to study the reactivity of the EEG burst suppression pattern to the EMF stimulation and 2) to study the changes in the EEG signal due to continuous high power EMF radiation.

## Materials and methods

**Figure 1:**
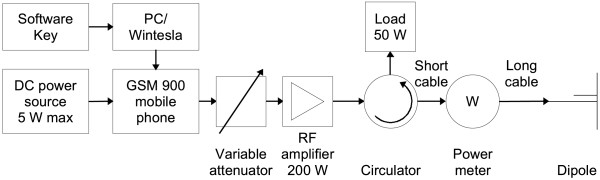
**Schematic diagram of the exposure system**.

**Figure 2:**
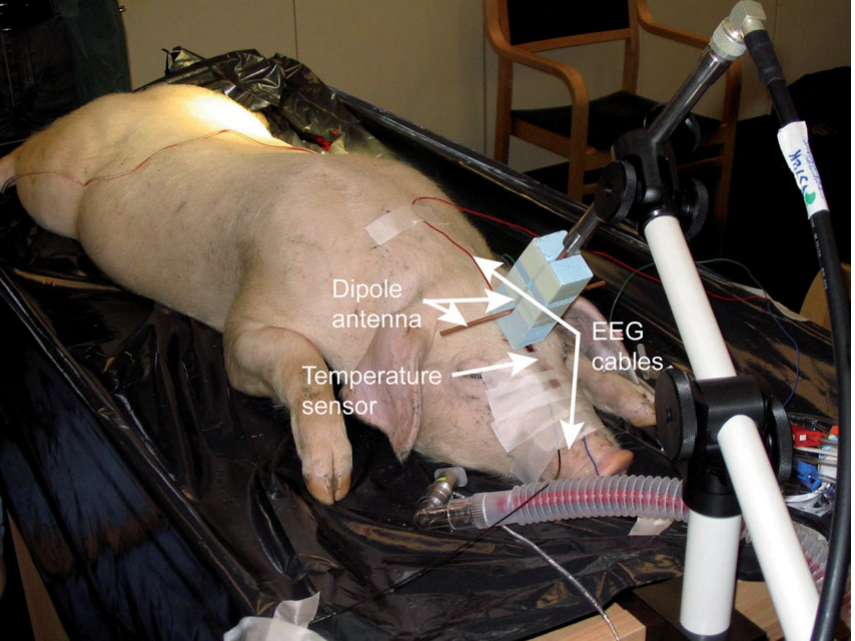
**Recording setup**.

Altogether 11 experiments were performed during the study. The experiments were carried out in two series with the first and the second experiment series including 5 and 6 animals, respectively. The animals were anaesthetized with isoflurane to the level where the burst suppression pattern occurs. The level of anaesthesia was controlled by visual inspection of the EEG signal in the course of the recordings. The first experiment series was recorded using Cadwell Easy Writer™ of Cadwell Laboratories, Kennewick, WA, USA (sampling frequency 200 Hz) and the second one with Biopac data acquisition system from Biopac Systems, Goleta, CA, USA (sampling frequency 1000 Hz). The exposures were conducted using a setup described in [26]. The exposure source was a half wave dipole fed by an amplified signal of a computer-controlled mobile phone. The mobile phone was transmitting a typical pulse modulated GSM voice call signal at 890.2 MHz (Figures 1 and 2). Each measurement consisted of two phases. During phase I the EMF stimulation was switched on and off in a random fashion while anaesthesia was kept at a level where the EEG suppression segments lasted 5 to 10 sec on the average. In phase II 10 min continuous stimulation was applied with the anaesthesia kept at a deep level with still continuous EEG. During the whole experiment the EEG, the electrocardiogram (ECG) and the stimulation marker were recorded. The EEG electrodes were implanted subcutaneously to obtain high quality signals. The functioning of the EEG and the ECG sensors in strong electromagnetic field was tested prior to the exposures by attaching the electrodes to a spherical dielectric phantom and irradiating it at maximum available power level (200 W peak). No interference was observed.

All animals received humane care in accordance with the Principles of Laboratory Animal Care formulated by the National Society for Medical Research and the Guide for the Care and Use of Laboratory Animals prepared by the Institute of Laboratory Animal Resources, National Research Council, and published by the National Academy Press, revised in 1996. The ethical evaluations of experiments were reviewed and approved by the Ethical Committee for Research Animal Care and Use of the University of Tampere.

### Phase I: reactivity of the burst-suppression pattern to EMF exposure

**Figure 3:**
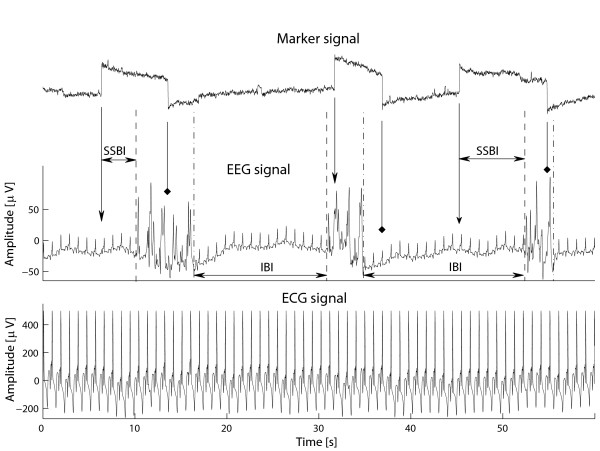
**A sample of the recorded signals of phase I recording**. The vertical arrows with V-shaped and diamond-shaped heads indicate the time instances of on- and off-switching the EMF source, respectively. Start- and end times of bursts are marked by dashed and dash-dotted lines, respectively. Three events, the endpoints of the first and the third stimulation period as well as the onset of the second stimulation period are discarded as they occur during bursts. Also, the endpoint of the second stimulation period is discarded because the EMF source is switched on again before a burst occurs. Two stimulation-start-to-burst intervals (SSBI) and two interburst intervals (IBI) are indicated in the figure. The EEG signal is contaminated by ECG artifact, however, the bursts can easily be detected by visual analysis. The ECG artifact was not present during the periods of continuous 10 minute exposure of phase II.

During the about 10–20 min period of phase I of the experiment the EMF stimulation source was switched on and off for 1–10 s in irregular intervals, first at the exposure level of 7.3 W/kg and then at 31 W/kg (maximum 10 g average [14]). A sample of the recorded signals is shown in Figure 3.

The EEG signal was analyzed visually to detect the onsets of bursts and suppression segments. Interburst intervals (IBI) were calculated and accepted for further analysis if their length remained below 100 seconds. The start- and end times of stimulation were identified from the marker curve. The stimulation start- and end times occurring during EEG bursts were discarded. Also, if after a stimulation onset the stimulation was switched off before an EEG burst started, the onset of this particular stimulation period was discarded. Correspondingly, if after a stimulation endpoint a new stimulation period was started before an EEG burst occurred the particular stimulation endpoint was discarded (e.g., the endpoint of the second stimulation period in Figure 3). For the remaining stimulation start- and endpoints the Stimulation Start to Burst Intervals (SSBIs) and Stimulation End to Burst Intervals (SEBIs) were calculated. The total number of detected IBIs and stimulation periods together with the number of IBIs, SSBIs and SEBIs considered valid for further analysis are given in Table 1.

**Table 1:** Statistics on interburst intervals (IBI), stimulation periods, stimulation start to burst intervals (SSBI) and stimulation end to burst intervals (SEBI).

**Recording nr.**	**Total nr of IBIs**	**Nr of IBIs shorter than 100 sec.**	**Nr of stimulation periods**	**Nr of valid SSBIs**	**Nr of valid SEBIs**
Set 1 Rec. 1	214	213	79	47	40
Set 1 Rec. 2	128	127	89	48	35
Set 1 Rec. 3	195	194	83	47	36
Set 1 Rec. 4	218	218	67	47	28
Set 2 Rec. 1	93	93	37	10	8
Set 2 Rec. 2	156	156	88	41	57
Set 2 Rec. 3	54	54	45	19	15
Set 2 Rec. 4	188	188	44	30	17
Set 2 Rec. 5	73	73	28	2	12
Set 2 Rec. 6	105	105	55	31	17

**Total**	**1424**	**1421**	**615**	**322**	**265**

### Phase II: 10 minute continuous exposure

Following phase I, after a short period of no exposure, phase II of the experiment was carried out by switching on the EMF source for 10 minutes at the SAR level of 31 W/kg. Anaesthesia was kept at lighter level at this phase so that the EEG was continuous with no burst-suppression pattern observed. The temperature was measured subcutaneously using a fluoro-optic sensor (Luxtron 750) and the temperature values were written down manually every minute during (and for several minutes after) the continuous exposure period. About 10–20 minutes after the EMF source was switched off the animal was sacrificed. Heart rate was obtained from the ECG signal off-line using a simple prefilter and compare-to-threshold algorithm. Due to some technical problems in the recordings, the analysis of phase II recordings could only be performed for the recordings of set 2.

Six segments of half a minute duration were extracted from the EEG signal at the following phases of the experiment: 1) just before stimulation onset, 2) just after stimulation onset, 3) in the middle of the stimulation period, 4) just before the end of the stimulation period, 5) just after the end of the stimulation period, and 6) five minutes post-stimulus. The signal segments were low-pass filtered and decimated 10 times, the new sampling frequency being 100 Hz. Relative power in the following EEG frequency bands was calculated: delta (0.5–4 Hz), theta (4–8 Hz), alpha (8–12 Hz), beta (12–20 Hz), and gamma (20–45 Hz). The power spectrum was calculated using autoregressive model parameters (Yule-Walker algorithm). In addition, spectral entropy [27], approximate entropy [28], Higuchi fractal dimension [29], and Lempel-Ziv complexity [30] were estimated from the EEG signal segments.

## Results

### EMF exposure effects on the EEG

**Figure 4:**
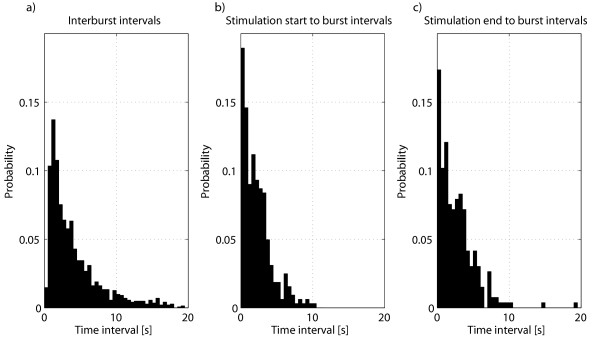
**Distributions of: a) interburst intervals, b) stimulation start to burst intervals and c) stimulation end to burst intervals**. 2.7% of the interburst intervals were longer than 20 seconds.

**Figure 5:**
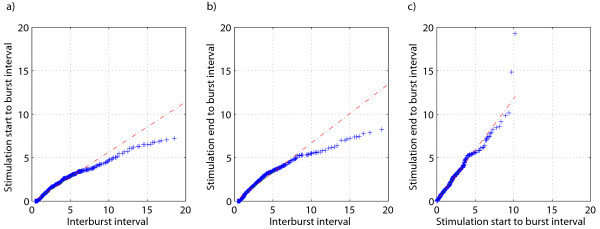
**Quantile-quantile plots of the distributions shown in Figure 4: a) stimulation start to burst interval vs. interburst interval, b) stimulation end to burst interval vs. interburst interval, c) stimulation start to burst interval vs. stimulation end to burst interval**.

By visual inspection of the EEG recordings of phase I, no obvious correlation between the timing of the burst-suppression pattern and the switching of the EMF source was observed. For better insight the distributions of IBIs, SSBIs, and SEBIs were plotted (see Figure 4). While the distributions of SSBIs and SEBIs look similar, very few IBIs shorter than 0.5 s occur. Also, the distribution of IBIs is wider, which comes from the fact that stimulation start- and end times coinciding with bursts were omitted. Mean IBIs, SSBIs and SEBIs were 4.76, 2.25, and 2.70 s, respectively. The shapes of the distributions of Figure 4 were further compared by obtaining quantile-quantile plots (see Figure 5). SSBIs and SEBIs follow very similar distributions (the right panel of the figure) if not taking into account a couple of outliers. The distribution of IBIs is more long-tailed compared to those of SSBIs and SEBIs, causing the data in the left and middle panels of Figure 5 to deviate from the straight line.

**Figure 6:**
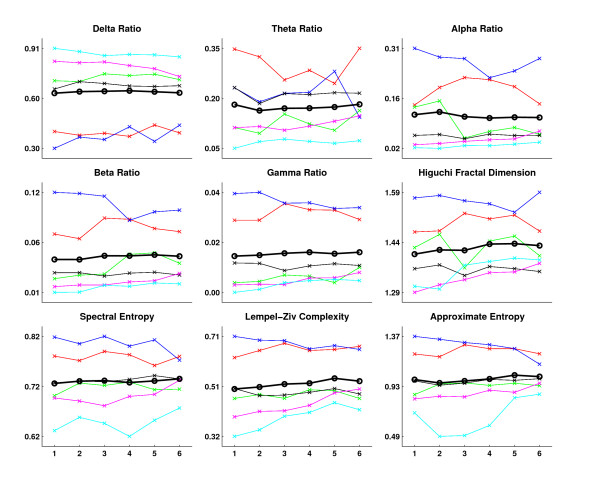
**The results of the EEG analysis of phase II recordings**. The curves correspond to the experiment animal recordings (recording 1: red, recording 2: green, recording 3: blue, recording 4: black, recording 5: magenta, recording 6: cyan). The black line with circle markers indicates the mean over the recordings. All the measures are dimensionless, indicating either power ratio or signal complexity in arbitrary units. The subsegments are numbered according to the time flow of the experiments – from pre-stimulus (segment 1) to 5 min post-stimulus (segment 6).

The results of the EEG analysis of phase II recordings are shown in Figure 6 and the statistical analysis is presented in Table 2. The results indicate that no significant changes can be observed in the calculated EEG measures due to the continuous EMF exposure. The recordings show quite different behaviour, however. In recordings 1 and 3 a clear peak in the power spectrum at around 7–9 Hz was observed. Also, the entropy measures were higher for these test animals. In some recordings certain measures like theta ratio (for recording 1) or approximate entropy (for recording 6) show clear correlation with the EMF exposure. However, these results do not repeat in other test animals.

**Table 2:** P-value obtained pairwise for all the calculated EEG measures and all the extracted segments using Wilcoxon ranksum test.

	**Delta**						**Theta**						**Alpha**				
	
	***seg2***	***seg3***	***seg4***	***seg5***	***seg6***		***seg2***	***seg3***	***seg4***	***seg5***	***seg6***		***seg2***	***seg3***	***seg4***	***seg5***	***seg6***
***seg1***	0.94	1.00	0.94	0.94	0.94		0.82	0.82	1.00	1.00	0.94		0.82	0.94	0.94	0.94	0.70

***seg2***		1.00	1.00	0.94	0.94			0.70	0.59	0.82	0.70			0.94	0.94	1.00	1.00

***seg3***			1.00	0.94	0.94				1.00	0.94	1.00				1.00	0.82	0.48

***seg4***				0.94	0.94					1.00	0.82					0.94	0.94

***seg5***					0.82						0.94						0.82

	**Beta**						**Gamma**						**HFD**				
	
	***seg2***	***seg3***	***seg4***	***seg5***	***seg6***		***seg2***	***seg3***	***seg4***	***seg5***	***seg6***		***seg2***	***seg3***	***seg4***	***seg5***	***seg6***

***seg1***	0.94	0.82	0.70	0.59	0.59		0.82	0.70	0.59	0.59	0.59		0.70	0.82	0.59	0.59	0.59

***seg2***		1.00	0.82	0.59	0.59			0.94	0.59	0.70	0.59			1.00	0.82	0.82	0.70

***seg3***			1.00	0.70	0.59				0.82	1.00	0.70				0.59	0.70	0.39

***seg4***				0.82	1.00					0.82	0.82					0.94	0.94

***seg5***					0.94						0.82						1.00

	**SPEN**						**LZC**						**APEN**				
	
	***seg2***	***seg3***	***seg4***	***seg5***	***seg6***		***seg2***	***seg3***	***seg4***	***seg5***	***seg6***		***seg2***	***seg3***	***seg4***	***seg5***	***seg6***

***seg1***	1.00	0.82	0.82	0.82	0.82		0.94	0.82	0.82	0.61	0.73		0.94	1.00	0.82	0.59	0.59

***seg2***		0.94	0.94	1.00	0.70			1.00	0.73	0.39	0.59			0.82	0.94	0.70	0.70

***seg3***			1.00	1.00	0.94				0.94	0.59	0.82				1.00	0.94	1.00

***seg4***				0.94	1.00					0.59	0.82					1.00	1.00

***seg5***					0.82						0.70						0.94

HFD – Higuchi Fractal Dimension, SPEN – Spectral Entropy, LZC – Lempel-Ziv Complexity, APEN – Approximate Entropy

### EMF exposure effects on heart rate and subcutaneous temperature

**Figure 7:**
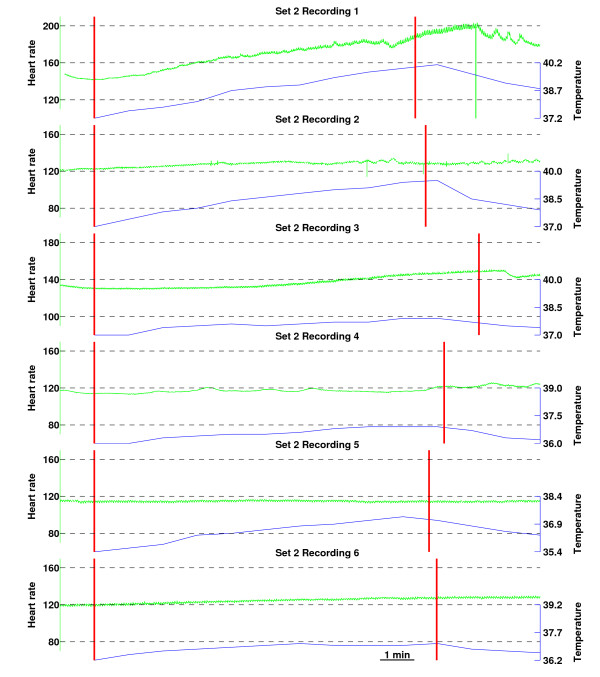
**Heart rate (green) and temperature (blue) changes during 10 minute exposure periods in phase II**. The time instances at which exposure was switched on or off are marked by vertical lines and the curves are aligned according to the onset of the exposure. The length of the exposure periods varied slightly from recording to recording. Temperature values were written down manually at every minute during (and for several minutes after) the exposure periods. While start- and endpoints of exposure periods are aligned accurately with the heart rate curve, there may be slight (up to half a minute) misalignment between heart rate and temperature.

Heart rate and temperature changes during the exposure periods for recordings of data set 2 are shown in Figure 7. The curves are aligned according to the onset of the exposure period. The time instances at which exposure is switched on or off were obtained from the marker signal and are thus accurately aligned with the heart rate curve. There may be slight misalignment (up to about half a minute) between the temperature curves and the heart rate, however, as the temperature values were marked manually. During the 10 minute stimulation the subcutaneous temperature increased by 1.63 (standard deviation 0.89, range 0.9...2.9) degrees. When the heart rate curves were fitted with straight lines over the stimulation period and the difference between the last and first values of the line were taken as the heart rate change during the stimulation, the average heart rate increase was calculated to be 14.15 (standard deviation 17.03, range 0.08...45.57) beats per minute. Although the heart rate and temperature increased during the exposure in all the recordings, no clear correlation between the magnitudes of these changes could be noted.

## Discussion

Burst suppression pattern in the EEG signal is generated in the cerebral cortex. It has been shown that the cortex, when isolated from deep structures, produces burst suppression. This is also reflected by the fact that in brain damage and focal status epilepticus, focal burst suppression is sometimes seen. Even burst suppression recorded from depth electrodes is likely to be of cortical origin [23]. The bursts induced by minor somatosensory, auditory or photic stimuli are likely triggered by brainstem, as are the bursts induced in ischemic brain damage. The triggering sensory information may, thus, originate from auditory, somatosensory or visual systems and our experiment tests, whether radiation can produce similar triggering sensory effect.

The hypothesis that switching the EMF stimulation on or off would induce bursts during deep burst suppression level anaesthesia was not confirmed by our experiments. The shape of the distribution of interburst intervals was similar to that of the distributions of time intervals from the start and endpoints of the stimulus to the burst onset. This similarity of the distributions was further confirmed by quantile-quantile plots. The main difference in the shapes of the distributions was the considerably higher probability of very short intervals from stimulus start/end to burst onset compared to IBIs. This can be explained by the physiological refractory period after bursts, making IBIs of less than 1 s improbable in the spontaneous burst suppression pattern. Also, during visual annotation of burst start- and end times short suppression segments (< 1 s) between bursts are often ignored. Omitting stimulation events during bursts might cause bias, but in our view this was the most reasonable way of handling these events. If there is already a burst going on while EMF source is switched on (or off), there is no way to tell if this particular switching had an influence on the bursting dynamics in the EEG.

The data recorded in this project does not give a comprehensive answer about the possible interaction between the EMF stimulation and the EEG bursting phenomenon as no sham recordings were available and the two phenomena, spontaneous bursting and possible interaction, could not be separated from one another. The similarity of the distributions indicates, however, that if such kind of interaction exists, it is not obvious.

The frequency content of the EEG signal showed no significant changes related to the continuous EMF stimulation in phase II recordings. Various measures of signal entropy and complexity have recently been found to correlate with changes in the EEG due to, for example, deepening anaesthesia [31]. However, none of the measures applied in this study: Higuchi fractal dimension, spectral entropy, Lempel-Ziv complexity, or approximate entropy, revealed any trends during the stimulation. This might be surprising as a clear increase in temperature by about 1–2 degrees was observed. A possible reason for these results might be that the temperature was measured from below the scalp while it is possible that increased blood perfusion has compensated for the potential temperature increase due to the EMF power in deeper brain structures. This explanation is supported by the increase in heart rate. Unfortunately, blood pressure was not recorded in the experiments. Also, other variables like the anaesthetic effect, for example, were not fully controlled.

## Conclusion

The exposure to 890 MHz EMF stimulation at 31 W/kg increased the subcutaneous temperature of the experiment animals by about 1.6 °C and the heart rate by 14.2 bpm in a population of six anaesthetised pigs. No significant changes were observed in the EEG activity of the pigs although several EEG signal analysis methods were applied. In a population of ten pigs, anaesthetized to the level where the EEG burst suppression pattern occurs, no correlation was found between switching on or off the EMF stimulation and triggering of the bursts.

In this study we wanted to see if RF radiation at high exposure levels could trigger the hypersensitive nonlinear burst suppression control system of anaesthesia EEG. However, in none of our pigs was there any sign of this kind of activation. The results of our study indicate therefore that the RF radiation does not produce sensory stimulation of somatosensory, auditory or visual system or directly affect the brain so as to produce EEG bursts during suppression. The slow rise in temperature at very high RF intensity suggests that there is an effect also in brain tissue, but this is not appropriate, intensive or abrupt enough to trigger the burst control system.

## Competing interests

The authors declare that they have no competing interests.

## Authors' contributions

MR and KA were responsible for anesthetizing the animals. AM performed the surgical procedures. VJ was responsible for recording the EEG. TT, TToivo and LK were responsible for the dosimetry, design and implementation of the exposure system. RF, TL, AV and VJ performed the data analysis. LK acted as the coordinator of the research team. The manuscript was compiled mostly by TL with the contribution of all the other authors. All authors read and approved the final manuscript.
